# Astym treatment *vs.* eccentric exercise for lateral elbow tendinopathy: a randomized controlled clinical trial

**DOI:** 10.7717/peerj.967

**Published:** 2015-05-19

**Authors:** Thomas L. Sevier, Caroline W. Stegink-Jansen

**Affiliations:** 1 Performance Dynamics, IU Health Ball Memorial Hospital, Muncie, IN, USA; 2 Department of Orthopaedic Surgery and Rehabilitation, The University of Texas Medical Branch, Galveston, TX, USA

**Keywords:** Astym, Lateral epicondylitis, Tendinitis, Tendinopathy, Tennis elbow, Treatment

## Abstract

**Introduction.** Patients with chronic lateral elbow (LE) tendinopathy, commonly known as tennis elbow, often experience prolonged symptoms and frequent relapses. Astym treatment, evidenced in animal studies to promote the healing and regeneration of soft tissues, is hypothesized to improve outcomes in LE tendinopathy patients. This study had two objectives: (1) to compare the efficacy of Astym treatment to an evidence-based eccentric exercise program (EE) for patients with chronic LE tendinopathy, and (2) to quantify outcomes of subjects non-responsive to EE who were subsequently treated with Astym treatment.

**Study Design.** Prospective, two group, parallel, randomized controlled trial completed at a large orthopedic center in Indiana. Inclusion criteria: age range of 18–65 years old, with clinical indications of LE tendinopathy greater than 12 weeks, with no recent corticosteriod injection or disease altering comorbidities.

**Methods.** Subjects with chronic LE tendinopathy (107 subjects with 113 affected elbows) were randomly assigned using computer-generated random number tables to 4 weeks of Astym treatment (57 elbows) or EE treatment (56 elbows). Data collected at baseline, 4, 8, 12 weeks, 6 and 12 months. Primary outcome measure: DASH; secondary outcome measures: pain with activity, maximum grip strength and function. The treating physicians and the rater were blinded; subjects and treating clinicians could not be blinded due to the nature of the treatments.

**Results.** Resolution response rates were 78.3% for the Astym group and 40.9% for the EE group. Astym subjects showed greater gains in DASH scores (*p* = 0.047) and in maximum grip strength (*p* = 0.008) than EE subjects. Astym therapy also resolved 20/21 (95.7%) of the EE non-responders, who showed improvements in DASH scores (*p* < 0.005), pain with activity (*p* = 0.002), and function (*p* = 0.004) following Astym treatment. Gains continued at 6 and 12 months. No adverse effects were reported.

**Conclusion.** This study suggests Astym therapy is an effective treatment option for patients with LE tendinopathy, as an initial treatment, and after an eccentric exercise program has failed.

**Registration/Funding.** Ball Memorial Hospital provided limited funding. Trial registration was not required by FDAAA 801.

**Known about the Subject.** Under the new paradigm of degenerative tendinopathy, eccentric exercise (EE) is emerging as a first line conservative treatment for LE tendinopathy. EE and Astym treatment are among the few treatment options aiming to improve the degenerative pathophysiology of the tendon. In this trial, Astym therapy, which has shown success in the treatment of tendinopathy, is compared to EE, which has also shown success in the treatment of tendinopathy.

**Clinical Relevance.** There is a need for more effective, conservative treatment options. Based on the current efficacy study, Astym therapy appears to be a promising, non-invasive treatment option.

## Introduction

Tennis elbow, or lateral elbow (LE) tendinopathy, is a common disorder that affects about 1–3% of the population (Croisier et al., 2007; Hoogvliet et al., 2013). Typical symptoms are pain at the lateral epicondyle of the humerus aggravated by loading of the common extensor tendinous origin of the forearm extensor muscles. The cause is primarily repetitive overuse, and heavy manual labor increases the risk of being affected (Peterson et al., 2014). Even though LE tendinopathy may present with a relatively uncomplicated clinical picture in many cases, the underlying pathophysiology exhibits a more complex state and the optimal management of LE tendinopathy has not been conclusively determined (Coombes, Bisset & Vicenzino, 2009). Numerous options for treatment are available, often with conflicting rationale and/or an absence of proof of principle for the proposed mechanisms of action. New treatment options continue to be investigated due to cases presenting with prolonged symptoms and frequent relapses (Kazemi et al., 2010).

Considering the history of a relatively poor understanding of the etiology and pathophysiology of LE tendinopathy, it is not surprising that optimal treatment methods are unclear. In recent years, there has been an increased understanding and a paradigm shift in the proposed pathophysiology of tendinopathy. Historically, the inflammatory model of tendinopathy was widely accepted, as it was believed to be a primarily inflammatory disorder and regularly referred to as tendinitis, with the suffix “itis” denoting inflammation (Khan et al., 2002). Histological studies have now revealed that tendinopathy and in particular LE tendinopathy, primarily involves a degenerative process (Khan et al., 1999; Nirschl, 1992). However, the overall absence of inflammation in tendinopathy, or whether it plays some role in its development, is still the subject of debate (Battery & Maffulli, 2011).

Many of the treatments for LE tendinopathy arose under the inflammatory model for tendinopathy and are still in use today, quite often with limited or absent scientific evidence supporting their effectiveness (Cullinane, Boocock & Trevelyan, 2014). A systematic review and meta-analysis of clinical trials on physical interventions for LE tendinopathy (Bisset et al., 2005), concluded that the literature has assessed the effect of a range of physical interventions on LE tendinopathy and failed to elucidate any long term beneficial effects over that of a placebo group.

A commonly used therapeutic intervention for LE tendinopathy is deep transverse friction massage (DTFM); however, a Cochrane review specifically examining DTFM in the treatment of tendinopathy concluded that DTFM did not significantly reduce tendinopathy symptoms compared to control treatment (Brosseau et al., 2002). In an update of this Cochrane review, it was noted that in tennis elbow, there is insufficient evidence to determine the effects of DTFM on pain, improvement in grip strength, and functional status, as no evidence of clinically important benefits was found (Loew et al., 2014). Instrument Assisted Soft Tissue Mobilization (IASTM) utilizes tools to perform DTFM (Miners & Bougie, 2011); however, the addition of tools does not appear to improve the effectiveness of DTFM. In a pilot study of 30 subjects with LE tendinopathy, IASTM was compared to no treatment (subjects waiting with information provided on stretching, ergonomics and what to expect with the condition), and it was found that IASTM results were comparable to no treatment being provided (Blanchette & Normand, 2011).

Therapeutic interventions that could impact the healing of the tendon include extracorporeal shockwave therapy (ESWT), therapeutic ultrasound and eccentric exercise. ESWT has been used in the treatment of soft tissue and bone-related musculoskeletal disorders for some time, however, the heterogeneous evidence base and the diversity of treatment types and protocols make interpreting the literature on its efficacy challenging. The proposed mechanisms for ESWT have the potential to address the underlying pathology of tendinopathy, and include direct effects on tissue calcification, alteration of cellular activity through cavitation, acoustic microstreaming, alteration of cell membrane permeability and effects on nociceptors through hyperstimulation blocking the gate control mechanism (Speed, 2014). Therapeutic ultrasound has been used in the treatment of tendinopathy, and although it may stimulate cell migration, proliferation, and collagen synthesis of tendon cells as indicated in animal studies (Tsai, Tang & Liang, 2011), there is conflicting evidence regarding its use in the treatment of musculoskeletal disorders (Andres & Murrell, 2008).

The conservative, therapy-based treatment that has been suggested to be the first-line of treatment for tennis elbow is eccentric exercise (Ackermann & Renström, 2012; Murtaugh & Ihm, 2013; Kaux et al., 2011; Orchard & Kountouris, 2011), which involves lengthening the musculotendinous unit while a load is applied to it. Eccentric exercise has demonstrated encouraging results, although the literature is limited, eccentric programs are varied, and optimal dosing has not yet been defined (Raman, MacDermid & Grewal, 2012).

The exact mechanisms by which eccentric exercises are effective in treating tendinopathy remain unclear (Maffulli, Longo & Denaro, 2010). There appears to be significant positive changes in tissue structure from eccentric exercise, but the clinical significance of these changes has not been determined (Knobloch et al., 2007; Langberg et al., 2007; Ohberg & Alfredson, 2004; Shalabi et al., 2004). In clinical trials, a program of eccentric exercises has demonstrated superior efficacy in the treatment of LE tendinopathy, as compared to therapeutic ultrasound (Selvanetti et al., 2003), bracing (Söderberg, Grooten & Ang, 2012), and multimodal rehabilitation programs consisting of: ice, therapeutic ultrasound, TENS, friction massage, and stretching (Croisier et al., 2007); and friction massage, ultrasound, heat, ice, and stretching (Tyler et al., 2010).

More recent treatments developed under the degenerative model of tendinopathy specifically target tendon healing and regeneration. Methods focusing on regeneration include regenerative injection techniques of autologous blood and plasma rich in platelets (Gosens et al., 2011), and the non-invasive rehabilitation process known as Astym^®^ therapy (Davies & Brockopp, 2010; McCormack, 2012; Slaven, 2014). (Performance Dynamics, Muncie, Indiana, USA)

Astym therapy is an emerging rehabilitation treatment that shares clinical reasoning with the regenerative injection techniques that aim to improve the pathologic structural changes of the tendon, while at the same time differing from these therapies by being non-invasive and a more comprehensive, rehabilitation approach to the affected tendon and surrounding tissues. A preparatory line of research preceded clinical trials to explore and substantiate the cellular impact of Astym treatment. Research on Astym treatment began with a multi-disciplinary research team of physicians, physiologists, therapists, cellular biologists and biomechanists, theorizing about a physical treatment method to potentially regenerate and remodel soft tissues, through possible activation of fibroblasts by means of the endogenous release of cellular mediators and growth factors. This activation, theorized to occur from stimulation by physical pressure and shear forces selectively applied topically and aimed at underlying tissues, would be strengthened when applied within the context of a rehabilitation program containing specific tendon loading and functional activity to guide the adaptation of tissues and optimize tissue quality. This theoretical reasoning has since been developed in proof of concept studies. Basic science studies were conducted on Astym treatment (Davidson et al., 1997; Gehlsen, Ganion & Helfst, 1999) to elucidate physiologically relevant mechanisms, and to develop specific treatment protocols aimed at stimulating the regeneration of soft tissues and the resorption of inappropriate scar tissue/fibrosis.

Guided by these basic science findings, specific protocols were developed defining the use of hand-held instrumentation to topically locate underlying dysfunctional soft tissue and then transfer particular pressures and shear forces to the dysfunctional tissue. The hand-held Astym instrumentation is designed to assess the presence of dysfunctional tissue by amplifying the tactile sensation of the underlying texture of the soft tissues in order to provide the treating clinician with indications where rough or improperly organized tissue is located. Once an area of potential dysfunctional tissue is located, the clinician applies appropriate pressures and shear forces to that tissue, aimed at initiating a reparative cellular response in dysfunctional tissue. *In vivo* studies revealed that the Astym protocols improved tendon repair, increased limb function, and normalized movement patterns in an animal model (Davidson et al., 1997; Gehlsen, Ganion & Helfst, 1999). Further, Astym treatment resulted in a significant increase in both fibroblast activation and fibroblast number, as well as the production of fibronectin, which together with interstitial collagens may interact to form a fibrillar component of the extracellular matrix (Davidson et al., 1997; Gehlsen, Ganion & Helfst, 1999). The increase in fibronectin is notable in that fibronectin is thought to be required for normal collagen organization and deposition by fibroblasts and they have the potential to guide cell and tissue behavior during healing as a function of their unique mechanical and bioactive properties (Branford et al., 2011; McDonald, Kelley & Broekelmann, 1982).

The standardized Astym treatment process contains three components in addition to the topical application of instruments in specific protocols: (i) the assessment and treatment of the entire kinetic chain to address fibrosis and dysfunctional soft tissue within the region. Fibrosis and dysfunctional soft tissue often occur in particular patterns associated with certain diagnoses, and those applying Astym treatment assess the patient to determine whether such a pattern is present, and if so, utilize Astym treatment on those areas; (ii) functional exercise components which include stretching and strengthening to properly load the tissues along longitudinal lines in order to promote healthy, functional alignment of new collagen deposition, and also to address the need for mechanical loading to extend and enhance regenerative properties of growth factors as shown in animal models (Davies & Brockopp, 2010; McCormack, 2012; Virchenko & Aspenberg, 2006); and (iii) the objective of detecting and reducing fibrosis that may be causing irritation or restrictions in movement. Astym treatment has been found to reduce pain and increase motion in cases where scar tissue was impeding movement (Davies & Brockopp, 2010).

After completion of the animal studies, Astym treatment has shown promise in the treatment of tendinopathy by demonstrating reduction in pain and increases in motion and functional ability (McCormack, 2012; Sevier & Wilson, 1999; Slaven, 2014; Wilson et al., 2000). Additional randomized clinical trials are needed to more fully investigate the efficacy of Astym treatment, and also to compare the efficacy of Astym treatment to other regenerative medicine approaches.

The primary objective of this study was to investigate the efficacy of the Astym treatment process as the initial treatment for patients with chronic lateral elbow tendinopathy as compared to an evidence-based eccentric exercise and stretching program (EE) based upon a program as suggested by Nirschl & Kraushaar (1996). The secondary objective of this study was to investigate the efficacy of Astym therapy in treating recalcitrant LE tendinopathy in patients who failed to resolve with the evidence-based eccentric exercise program.

## Methods

### Design

**Figure 1:**
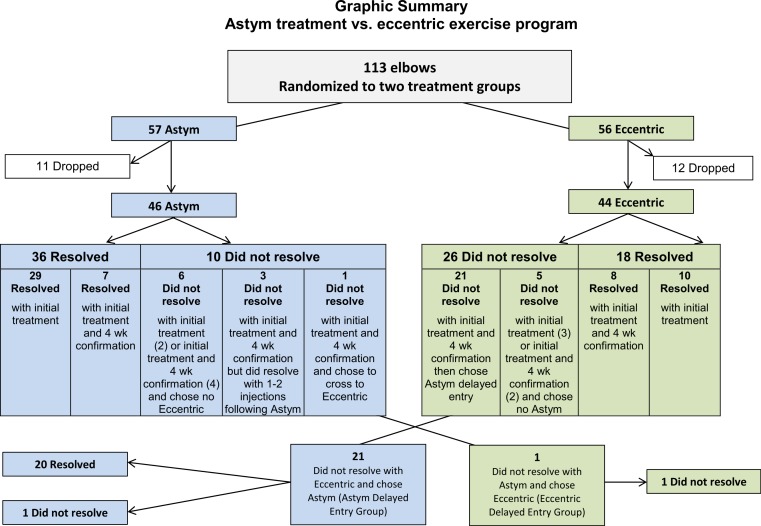
Flow sheet of number subjects enrolled, retention and drop-outs through the course of the study.

The study had a prospective, two group, and parallel randomized controlled trial design. Randomization was performed using computer-generated random number tables. The controlled trial of four weeks of treatment was conducted followed by a four week period of no treatment intervention (“confirmation period”), in which the results of the treatment were allowed to fully manifest or dissipate. Following the controlled trial phase of this study and the confirmation period, subjects who did not resolve their symptoms were allowed to opt to receive the other treatment in the explorative secondary portion of this study for recalcitrant subjects (Delayed Entry Groups). All subjects received follow-up questionnaires at 6 and 12 months after the initial baseline visit. Figure 1 shows the design of the study with the number of subject elbows listed in each portion of the study. Table 1 shows the randomized group assignment and demographic and baseline comparisons.

**Table 1:** Demographic and baseline comparisons between the eccentric exercise group and the Astym treatment group. Reported are the numbers of subjects, mean and in parentheses the standard deviations of the measures scores.

Treatment groups	Eccentric exercise program	Astym treatment	Group difference (level of significance *p* < 0.05)
Number of subjects	53	54	*p* = 0.46
Number of elbows	56	57	*p* = 0.93
Number of drop-outs	12	11	*p* = 0.40
Number of elbows following drop-outs	44	46	*p* = 0.40
Gender	39 females;	23 females;	*p* = 0.04
	14 males	31 males	*p* = 0.01
Age initial	46.3 (SD 7.2)	47.6 (SD 5.9)	*p* = 0.32
Age Drop-out subjects	41.7 (SD 7.7)	43.2 (SD 4.6)	*p* = 0.62
**Primary outcome measure**			
DASH (0 → 100)	29.8 (SD 14.6)	29.5 (SD 14.5)	*p* = 0.93
**Secondary outcome measures**			
VAS Act (in mm)	53 (SD 25)	59 (SD 24)	*p* = 0.21
Max grip (in lbs force)	58.1 (SD 30.8)	70.3 (SD 35.1)	*p* = 0.062
Function (in mm)	62 (SD 25)	59 (SD 25)	*p* = 0.055

**Notes.**

mm Millimeters lbs American pounds

The rater taking the measurements from the subjects was blinded to the group allocation, as well as the physicians evaluating the recovery of the subjects. The patients, office staff, treating therapists and the study coordinator were instructed not to reveal the type of physical therapy intervention to the physicians. The subjects and the treating clinicians could not be blinded due to the nature of the treatments. At 8 weeks, after 4 weeks of treatment and a confirmation period of no treatment intervention for 4 weeks, subjects with unresolved symptoms in both groups had the option to cross over to the alternate treatment group for additional treatment with a duration of 4 weeks (Delayed Entry Groups).

Subjects received physician assessment at baseline and reassessments after each four week period until they met resolution criteria, at which time they were discharged from treatment and on-site assessments. Determination of resolution was made using a 15 point Patient and Physician Global Rating of Change Scale (GRC) ranging from −7 (a very great deal worse) through 0 (no change) to +7 (a very great deal better) that has used in a variety of settings to measure overall patient improvement (Fritz & Irrgang, 2001; Jaeschke, Singer & Guyatt, 1989; Kamper, Maher & Mackay, 2009). With equal weighting, a combined patient and physician score of eight or better was considered “resolved” for the purposes of this study. Subjects with combined scores less than eight continued in the treatment process. The physicians were blinded to the rating provided by the patient. Patients judged symptom resolution in comparison to symptoms experienced in the first clinic visit; the physician used clinical examinations to assess the signs of tendinopathy, such as direct palpation over the insertion of the tendon and pain with resisted wrist extension and middle digit extension, in combination with assessing the symptoms expressed by the patient through questioning as their basis for the GRC. In their review of global rating scales, Kamper, Maher & Mackay (2009) report acceptable rater agreement of 87% and acceptable inter-rater reliability (ICC (2, 1)) of 0.74.

There was no charge to the subjects for physician assessments and rehabilitation treatment associated with the study protocols. Subjects received $25 compensation for completion of each of the 6 and 12 month questionnaires ($50 total). The Ball Memorial Hospital Institutional Review Board, Muncie, Indiana, granted human subjects and written informed consent approval in writing, and informed consents were obtained from all subjects (IU Ball Memorial Health Institutional Review Board, BMH Study #389). Participants of the study were enrolled and the study was conducted prior to general recommendations that trials be registered, and registration of this trial was not required under Section 801 of the Food and Drug Administration Amendments Act.

### Subjects

Subjects were recruited through assistance of physicians and advertisement in the local media. Study eligibility criteria consisted of age between 18 and 65 years old with lateral elbow pain of greater than 12 weeks duration, and at least two of the following: pain on palpation of the lateral extensor muscle mass and/or lateral epicondyle area, pain at the lateral epicondyle on resisted wrist extension with the elbow fully extended, and pain at the lateral epicondyle with passive wrist flexion with the elbow fully extended. Exclusion criteria were history of systemic connective tissue diseases or polyarthralgia, history of cervical radiculopathy, degenerative disease of the cervical spine, history of corticosteroid injection in prior six weeks, history of regular use (one/day) of NSAIDs in prior one week, lack of full range of motion of the involved elbow, abrasion or direct trauma to the lateral elbow area, or abnormal antero-posterior and/or lateral X-rays.

Table 1 shows the enrollment data and baseline measures for the subjects as well as the drop-out information. A total of 107 subjects (113 elbows) were enrolled in the study with a mean age of 46.9 years old (SD 6.6 years). There were no significant differences between the two groups with regard to demographics and baseline measures, with the exception of gender (*p* < 0.002). The EE group had a majority of females enrolled, and in the Astym treatment group the majority of participants were male. Sample size estimates were calculated for the primary outcome measure, the Disability of the Arm Shoulder and Hand scale (DASH), for two effect sizes: a medium effect size of 0.58 and also for a large effect size of 0.8 (Murtaugh & Ihm, 2013). Based on expected effect sizes for the DASH of 0.5 and 0.8, a desired power of 0.8 and a level of significance of 0.05, the required sample size was estimated at 64 subjects and 26 subjects per group, respectively.

Figure 1 is a flowchart showing the numbers randomized subject allocation, actual subject participation and drop-outs. Subjects were dropped from the study if they did not return for measurement after baseline measurements. One hundred seven subjects (with 113 involved elbows) who met the eligibility criteria participated in the study and were randomized into the two study groups using computer-generated random number tables. If a patient qualified for the study with both elbows involved, the first elbow was randomly assigned to a group, and the second elbow was also assigned to the same group. The Astym treatment group included 57 elbows and the eccentric exercise (EE) group included 56 elbows. Following dropout, there were 46 elbows in the Astym treatment group and 44 elbows in the EE group.

The study took place at a large orthopedic center in Indiana. Both groups received medical assessments by, or under the supervision of, the lead investigator, S Craig Veatch, MD. The Astym treatment group and the EE group were seen by different clinicians for the therapy interventions. The EE group received exercise instruction in an outpatient therapy clinic of a large hospital, and then performed their exercise program at home. The Astym treatment group received their treatment and performed some exercise in the therapy clinic associated with the orthopedic center, and then performed additional exercise at home. One clinician, who had completed the Astym educational program and required testing for certification, performed the Astym treatment protocol on all subjects in the Astym treatment group. The subjects in the EE group received instructions by a different clinician who was not Astym certified. The two treating clinicians met prior to the study to review eccentric exercise and strengthening instructions to ensure consistency of instruction to both groups. All subjects were given written copies of exercise instructions.

### Interventions

#### Evidence-based eccentric exercise and stretching group (EE) (control group)

Subjects randomized to the EE group performed stretching and eccentrically focused strengthening exercises at home expanding on the program suggested by Nirschl & Kraushaar (1996). Stretching exercises were performed three times daily and eccentrically focused strengthening exercises were performed twice weekly. The stretching exercises consisted of a wrist flexor stretch with elbow extended, a wrist extensor stretch with elbow extended, a forearm wind-up stretch, and a triceps stretch. The forearm wind-up stretch exercise requires one arm crossed over the other, incorporating wrist palmar flexion and rotation in a pronatory motion to stretch the structures coming off the lateral epicondyle including the supinator muscle, as well as the brachioradialis and extensor carpi radialis longus. Subjects were instructed to perform the stretches three times daily for one repetition of 30 s each to the point of pull, but not to the point of pain.

The eccentric strengthening exercises consisted of six total exercises for the elbow, wrist, and shoulder and were to be performed 2 times per week for 2 pain free sets of 15 repetitions each, increasing to 3 sets as tolerated. Emphasis was made on the eccentric portion of the strengthening exercises. Progressive resistance was accomplished according to patient tolerance using increasing forces provided by Thera-Band^®^ (The Hygienic Corporation, Akron, Ohio, USA). Subjects were given initial instruction by a clinician and were provided written guidelines for exercise progression. If subjects had questions about exercises or progression of exercises, they were encouraged to call or meet with the instructing clinician for clarification. In addition, the treating clinician initiated contact with the subjects during the treatment period to answer questions and provide guidance on the EE program. No concerns regarding compliance or performance were noted. Effectiveness of eccentric exercise in tendinopathy has been documented in the literature both in terms of clinical improvement and positive changes in tissue structure (Murtaugh & Ihm, 2013; Raman, MacDermid & Grewal, 2012). A four-week program of eccentric exercise has been shown to improve VAS scores in patients with lateral elbow tendinopathy, many of whom had failed previous treatments of physical therapy, medication, and injection (Manias & Stasinopoulos, 2006), and was found to be significantly effective in reduction of pain and in the improvement of functional status in patients with lateral elbow tendinopathy (Viswas, Ramachandran & Korde Anantkumar, 2012).

#### Astym treatment group (Astym) (experimental group)

**Figure 2:**
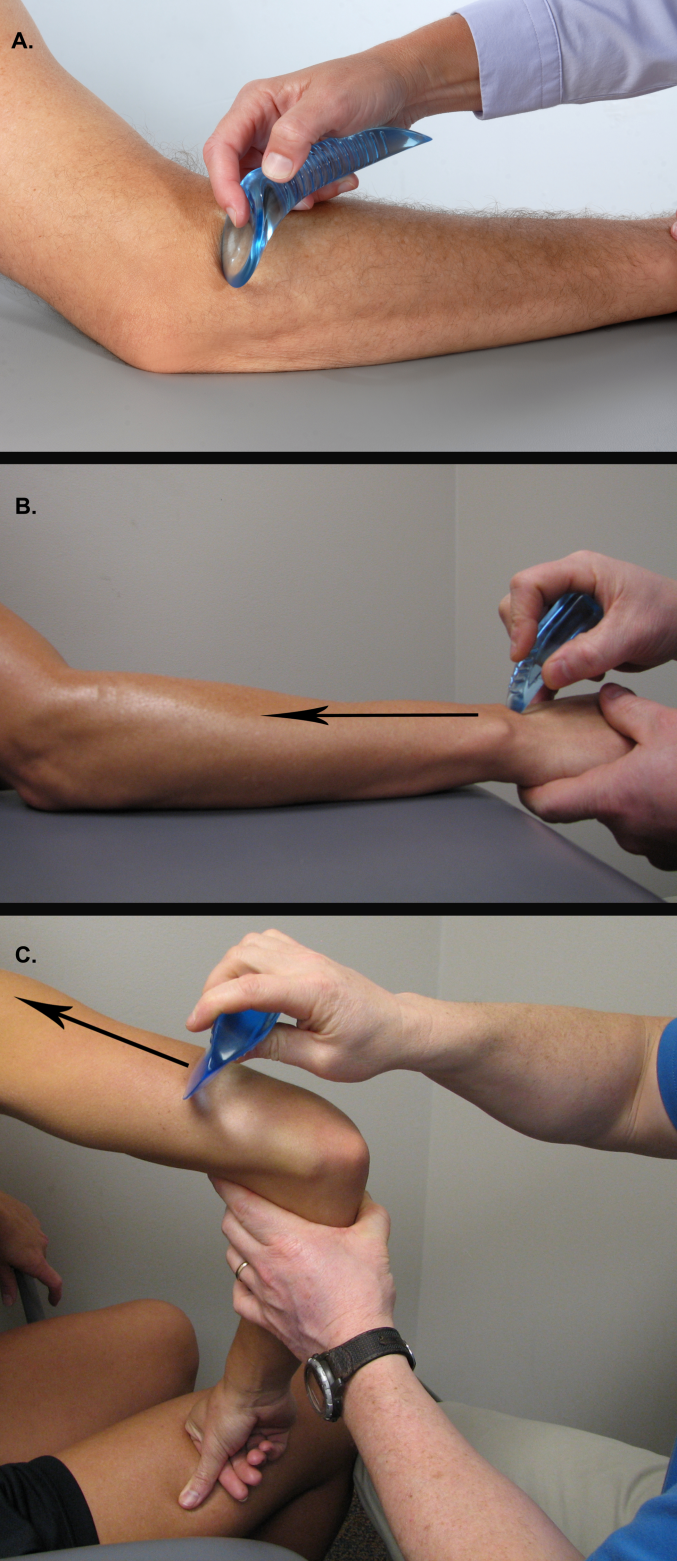
Astym treatment. (A) Astym treatment of tendinopathy of the lateral elbow. (B) Astym treatment of tendinopathy of the lateral elbow, distal kinetic chain. (C) Astym treatment of tendinopathy of the lateral elbow, proximal kinetic chain

Subjects assigned to the Astym treatment group were seen in a physical therapy clinic two times weekly for four weeks. At least two days were given between sessions to allow for adequate response to the theorized regenerative stimulation from Astym treatment. An Astym certified clinician performed the Astym treatment protocol on all subjects in the Astym treatment group. The treatment was performed by applying instruments topically in a systematic pattern throughout the involved extremity in order to deliver particular pressures and shear forces to the underlying tissue (Figs. 2A–2C). The standard Astym upper extremity treatment protocol was completed: (1) treatment of the affected arm from the wrist retinaculum to the deltoid on both the palmar and dorsal sides of the arm, and (2) stretching and eccentric strengthening components as part of Astym treatment, supervised by the clinician. In order to minimize any outcomes differences due to exercise selection, the Astym treatment and EE group received the identical stretching and eccentric strengthening exercise instruction. Stretching exercises were performed three times per day and eccentric strengthening exercises were performed twice weekly. The Astym treatment group performed their strengthening exercises within their twice weekly Astym treatment sessions while the EE group performed them at home. Both groups used the same exercises and guidelines for advancement to exercises with a higher intensity. Both groups were instructed that during the first two weeks of the study, they were allowed to take 200 mg of over-the-counter ibuprofen as needed for pain.

### Measurements and outcomes

Data were collected from all subjects during office visits at baseline and 4 weeks. After the 4 week mark, continuation of participation was dependent upon whether the subject met resolution criteria. If resolution criteria were not met, the participant continued to be actively enrolled in the study. Data were collected at 8 weeks from subjects who did not meet resolution criteria at 4 weeks, and at 12 weeks from subjects who did not meet resolution criteria at 4 and 8 weeks. Measurements at each office visit included the primary outcome measure, the DASH, and the secondary outcome measures, a 100 mm Visual Analog Scale (VAS) for pain with activity (0, no pain; 100, worst possible pain), a 100 mm Visual Analog Scale (VAS) for function (0, no use of the hand/wrist/arm; 100 normal use of the hand/wrist/arm), and maximum grip strength measured with the elbow extended. All of these measures are commonly used to assess treatment effects for tendinopathy of the lateral elbow (Dworkin et al., 2012; Maffulli, Longo & Denaro, 2010; Ruyssen-Witranda, Tubacha & Ravauda, 2011; Stratford et al., 1987).

The DASH has been shown to be a valid and reliable tool to measure patient perceptions of disability when affected upper extremity disorders (Beaton et al., 2001). The DASH has shown a responsiveness with large effect sizes (>0.8) in patients with wrist and hand injuries and a standardized response mean of 1.37 (Raman, MacDermid & Grewal, 2012). The VAS for pain is one of the most commonly used scales to document patient progress for a wide variety of patient populations (Litcher-Kelly et al., 2007; Scudds, 2001). In a review of measures used to assess chronic musculoskeletal pain in clinical and randomized controlled clinical trials, 60% of studies used a VAS to assess pain (Litcher-Kelly et al., 2007). A vertical 100 mm VAS scale has been shown to be valid and reliable as a measure of pain for patients with lateral epicondylopathy (Stratford et al., 1987; Waugh et al., 2004), as well as induced lateral elbow pain (Slater et al., 2006). The minimum clinically significant change in acute pain on the VAS pain scale has been shown to be 13 mm (Gallagher, Lievman & Bijur, 2001; Todd et al., 1996), although whether this applies to both acute and chronic conditions is unclear. (Ruyssen-Witranda, Tubacha & Ravauda, 2011; Stratford et al., 1987) investigated reliability and validity of commonly used outcome measures for lateral elbow tendinopathy. Those reliability estimates support the reliability of the measures used in this study: reliability of the vertical visual analog pain scale (ICC 0.89); for the vertical visual analog function scale (ICC 0.85) and for pain free grip strength (ICC 0.87); with maximal grip strength (ICC 0.6). All measures in this study showed a more than 56% increase in patients with successful recovery with the exception of maximum grip strength, which was used to measure changes over time (Stratford & Levy, 1994).

## Data Analysis

The data analysis consisted of two components: (1) analysis of the primary experimental study objective assessing the randomized group differences, (2) analysis of the explorative secondary study objectives, the longitudinal changes in outcome measures of subjects recalcitrant to the EE program who subsequently opted for Astym treatment, and the long term outcome differences between those who received Astym treatment early or late.

***1. Randomized experimental study objective analysis***

The data were screened for differences in baseline measures for the two groups (Table 1). Kolmogorov–Smirnov normality tests were applied to the used gain score parameters to ensure that requirement for the normality of distribution was met. Independent *t*-tests were performed comparing the gain scores for the EE group versus the Astym treatment group for the primary outcome measure, the DASH, and the secondary outcome measures, VAS with activity, VAS function and maximum grip strength. The primary outcome measure, the DASH, was tested at the level of significance of 0.05. Bonferroni adjustments were made to the secondary measures, and the secondary measures were tested at a level of significance of 0.05/3 = 0.017. Data were analyzed in two ways: using only participants who had received the assigned treatment and secondly using an intent-to-treat analysis imputing the total group mean to replace missing data, and they provided the same results. Effect sizes and power calculations were completed for the performed statistical analyses based on the true participant analyses.

The only baseline difference between the two groups was that the EE group contained a higher number of women. The impact of gender on the results was statistically tested as a follow-up analysis using an ANOVA analysis with two factors: the initial treatment and gender. Gender did not impact the results. Subjects in the Astym group performed their exercise program an average of 6.57 times per week, and the subjects in the EE group performed their exercise program an average of 6.19 times per week.

***2. Secondary explorative analyses: subjects not responding to EE who opted to receive Astym treatment (Astym Delayed Entry Group) (Table 4)***

Changes over time (scores at baseline, four weeks, eight and twelve weeks) of the Astym Delayed Entry Group subjects were analyzed using one way repeated measures ANOVA and contrast analyses (contrasting baseline and four weeks, four and eight weeks and eight and 12 weeks measurements).

To answer the question if recalcitrant subjects could achieve similar gains as obtained by subjects receiving Astym therapy as an initial treatment, one sample *t*-tests were performed to test the differences between the mean gain scores of the initial Astym treatment group of the experimental study phase and the Astym Delayed Entry Group. Gain scores were calculated for the Astym Delayed Entry Group for the period when Astym treatment was provided (post-test 2 at 8 weeks and post-test 3 at 12 weeks).

***3. Long term outcomes***

Long term results for all subjects were tested with paired *t*-tests comparing baseline scores to 6 months and 12 months scores. In addition, long term differences in gains made by the early Astym treatment and Delayed Entry Astym treatment subjects were tested using independent t-tests of the gain scores (baseline and six month and baseline and 12 month scores).

## Results

In the randomized clinical trial, 78.3% (36/46) of elbows in the Astym group responded by meeting resolution criteria from the initial treatment either at 4 weeks or at 8 weeks following the confirmation period. In the other group of the initial randomized clinical trial, 40.9% (18/44) of elbows in the eccentric exercise group (EE) met resolution criteria at 4 weeks or at 8 weeks following the confirmation period. In the explorative portion, one subject in the Astym group who did not resolve opted to receive EE, which did not result in resolution of symptoms. Therefore, no group results can be included for unresolved Astym patients, who opted to cross over to an eccentric exercise program. Of the 26 non-responders from the EE group, 21 subjects opted to receive Astym treatment (Astym Delayed Entry Group). Of these 21 recalcitrant EE non-responders, 20 (95.7%) met resolution criteria following a four week period of Astym treatment. Of the total number of 67 cases who were treated with Astym therapy (either initial or delayed) 56 cases (83.6%) met resolution criteria. No dropouts were reported due to failed response and no adverse effects were reported in either group. Both types of analyses, the true participant and intent-to-treat analyses, provided the same results. True participant results are reported below. Effect sizes and power calculations were completed for the performed statistical analyses based on the true participant analyses as well.

***1. Experimental analysis: randomized treatment comparisons of gain scores of Astym subjects versus EE subjects at 4 weeks (Tables 2 and 3)***

**Figure 3:**
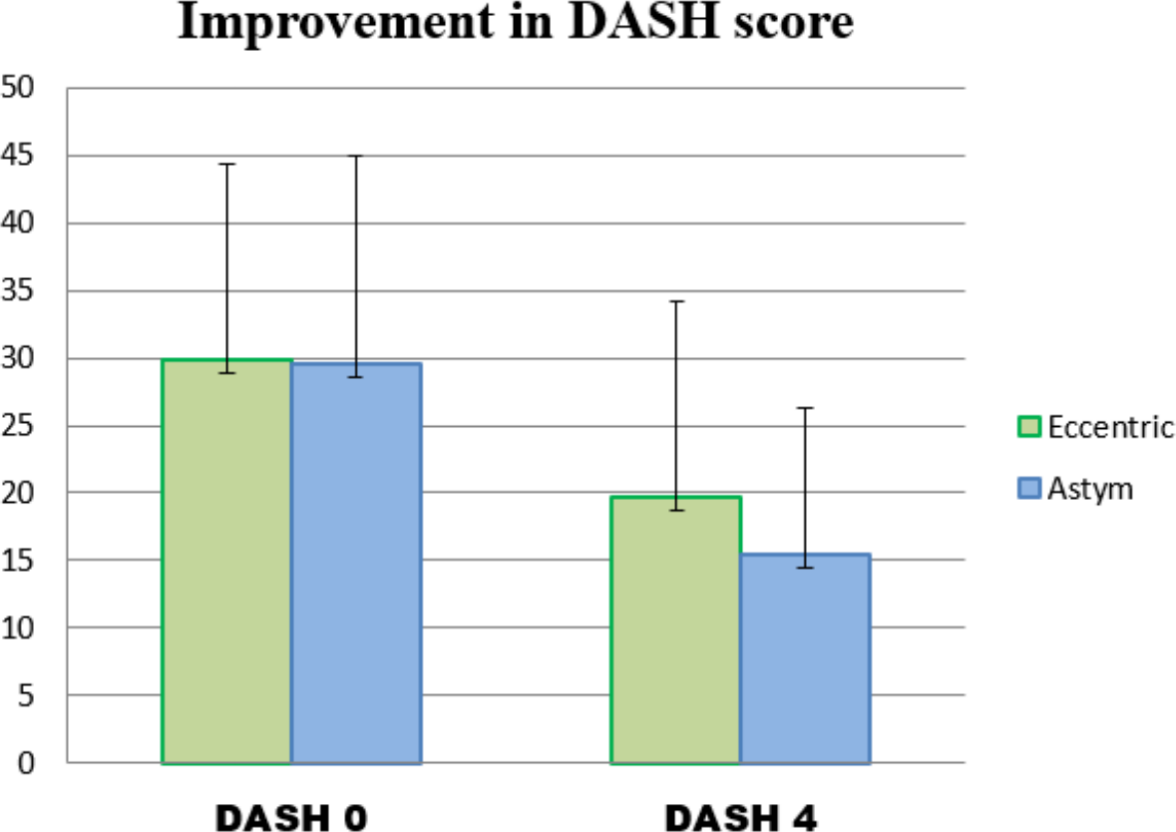
Mean and standard deviations of the DASH scores of the Eccentric and Astym Groups at Baseline (DASH 0) and at the closure of the 4 week randomized phase of the study (DASH 4).

The results of the independent t-tests applied to the primary and secondary outcome measures are shown in Table 2. Figure 3 shows the mean and standard deviations of the baseline and post treatment DASH scores (the primary outcome measure) from which the gain scores were calculated. Subjects who received Astym treatment had greater reductions in disability (DASH scores) (*p* = 0.047) than subjects in the EE group. Of the secondary outcome measures, Astym treatment subjects demonstrated greater gains in maximum grip strength (*p* = 0.008). There were no statistically significant differences between the two groups in pain with activity (*p* = 0.08) and function (*p* = 0.55).

**Table 2:** Randomized phase differences in gain scores for Astym vs. EE, calculated using baseline score minus four week score. Reported are the means and std deviations in parentheses, *p* values and effect sizes.

Comparison of gain scores	Eccentric exercise group	Astym treatment group	Significance	Effect size (95% confidence interval)	Power
**Primary outcome**					
Gain DASH (0 → 100)	↓7.8	↓13.3	*t* = − 2.0	0.42	*n* = 90
	(13.2)	(12.9)	*p* = 0.047	(0.00, 0.84)	power:50%
**Secondary measures**					
Gain Pain with activity (VAS)(in mm)	↓13 (28)	↓24 (27)	*t* = − 1.8	0.40	*n* = 80
			*p* = 0.08	(−0.05, 0.84)	power:43%
Gain Maximum grip strength (lbs)	↓1.9	↑9.4	*t* = 2.7	0.62	*n* = 79
	(15.9)	(18.5)	*p* = 0.008	(0.16, 1.07)	power:78%
Gain Function (VAS) (in mm)	↑12(28)	↑15(28)	*t* = 0.6	0.14	*n* = 75
			*p* = 0.55	(−0.31, 0.60)	power: 9%

**Notes.**

DASH, a decrease (↓) means less disability.

Function, an increase means greater functional ability.

**Table 3:** Mean, std deviations in (), number of subjects for the primary and secondary measures of the randomized phase, with short and long term f/u. Recalcitrant EE subjects allowed to choose Astym at 8 wks.

	DASH EE	DASH Astym	VAS active (mm) EE	VAS active (mm) Astym	Function (mm) EE	Function (mm) Astym	Max grip (lbs) EE	Max grip (lbs) Astym
	**Randomized phase**							
Baseline	29.8 (14.6)	29.5 (14.5)	53 (25)	59 (24)	62 (25)	59 (25)	58.1 (30.8)	70.3 (35.1)
	*N* = 56	*N* = 57	*N* = 50	*N* = 52	*N* = 45	*N* = 50	*N* = 48	*N* = 55
4 weeks	19.7 (11.4)	15.4 (10.9)	41 (24)	37 (25)	73 (24)	76 (21)	60.1 (35.9)	80.1 (33.5)
	*N* = 44	*N* = 46	*N* = 44	*N* = 44	*N* = 43	*N* = 45	*N* = 42	*N* = 43
Gain scores 0–4 weeks	↓7.8(13.2)	↓13.3(12.9)	↓13.3(28)	↓24(27)	↑12(28)	↑16(28)	↓1.9(15.9)	↑8.9(18.6)
	*N* = 44	*N* = 46	*N* = 40	*N* = 40	*N* = 35	*N* = 40	*N* = 38	*N* = 41
	**Short term follow-up**							
8 weeks	19.4 (12.1)	17.3 (12.7)	38 (25)	43 (29)	73 (22)	78 (23)	57.8 (31.6)	68.2 (38.2)
	*N* = 30	*N* = 15	*N* = 29	*N* = 14	*N* = 30	*N* = 14	*N* = 28	*N* = 13
12 weeks	9.3 (6.7)	NA	17 (19)	NA	85 (15)	NA	62.7 (33.8)	NA
	*N* = 18	*N* = 1	*N* = 19	*N* = 1	*N* = 19	*N* = 1	*N* = 17	*N* = 1
	**Long term follow-up**							
6 months	5.9 (6.8)	6.8 (5.9)	7 (10)	12 (14)	95 (8)	93 (13)	Not tested	Not tested
	*N* = 35	*N* = 33	*N* = 34	*N* = 31	*N* = 35	*N* = 33		
12 months	4.1 (6.7)	3.7 (4.6)	4 (7)	9 (18)	95 (10)	95 (10)	Not tested	Not tested
	*N* = 35	*N* = 30	*N* = 33	*N* = 29	*N* = 33	*N* = 29		

**Table 4:** Results from Astym Delayed Entry Group Astym treatment was provided starting at 8 weeks to recalcitrant eccentric subjects who completed their eccentric exercise program but continued to have unresolved symptoms and then opted to receive Astym treatment.

Measure	0–4 weeks eccentric	4–8 weeks (no treatment)	8–12 weeks astym delayed entry
**Primary outcome measure**			
DASH	Decrease 0.64%	Decrease 0.60%	Decrease 13.43%
	*F* = 0.07; *p* = 0.79	*F* = 0.17; *p* = 0.69	*F* = 35.81; *p* < 0.005
	Effect size 0.004	Effect size 0.01	Effect size 0.7
	Power 0.06	Power 0.07	Power 1.00
**Secondary outcome measures**			
Pain with activity (VAS)	Decrease 3.1 mm	Decrease 3.1 mm	Decrease 26.1 mm
	*F* = 0.22; *p* = 0.64	*F* = 0.41; *p* = 0.53	*F* = 12.91; *p* = 0.002
	Effect size 0.01	Effect size 0.03	Effect size 0.45
	Power 0.07	Power 0.09	Power 0.92
Maximum grip strength	Decrease 12.1 lbs	Increase 2.8 lbs	Increase 6.3 lbs
	*F* = 10.46; *p* = 0.006	*F* = 0.31; *p* = 0.59	*F* = 1.10; *p* = 0.31
	Effect size 0.42	Effect size 0.02	Effect size 0.07
	Power 0.85	Power 0.08	Power 0.17
Function (VAS)	Increase 13 mm	Decrease 1.2 mm	Increase 17 mm
	*F* = 3.59; *p* = 0.08	*F* = 0.05; *p* = 0.83	*F* = 11.3; *p* = 0.004
	Effect size 0.14	Effect size 0.003	Effect size 0.41
	Power 0.34	Power 0.06	Power 0.88

**Notes.**

Shaded areas are statistically significant improvements in the Astym Delayed Entry Group.

The percentage gain (the percentage expressed as the difference between the baseline and 4 week score divided by the baseline score) of the Astym treatment group was 47.9% for the primary outcome measure, the DASH. The secondary measures showed percentage gains of 37.8% for pain with activity (VAS), 28.5% for function (VAS), and 14.0% for maximum grip strength. The percentage gain of the EE group was 34% for the primary outcome measure, the DASH. The secondary measures showed percentage gains of 22.7% for pain with activity (VAS) and 17.0% for function (VAS), and a loss of 3.5% for maximum grip strength.

A follow-up examination regarding impact of gender showed no interaction effects between gender and initial treatment for the gain scores of the DASH (*p* = 0.19), maximum grip strength (*p* = 0.56), pain with activity (*p* = 0.12), and function (*p* = 0.24), indicating gender did not impact the results.

**2. *Explorative analyses: subjects recalcitrant to EE who received Astym treatment after week 8, Delayed Entry Group (Table 4)***

**Figure 4:**
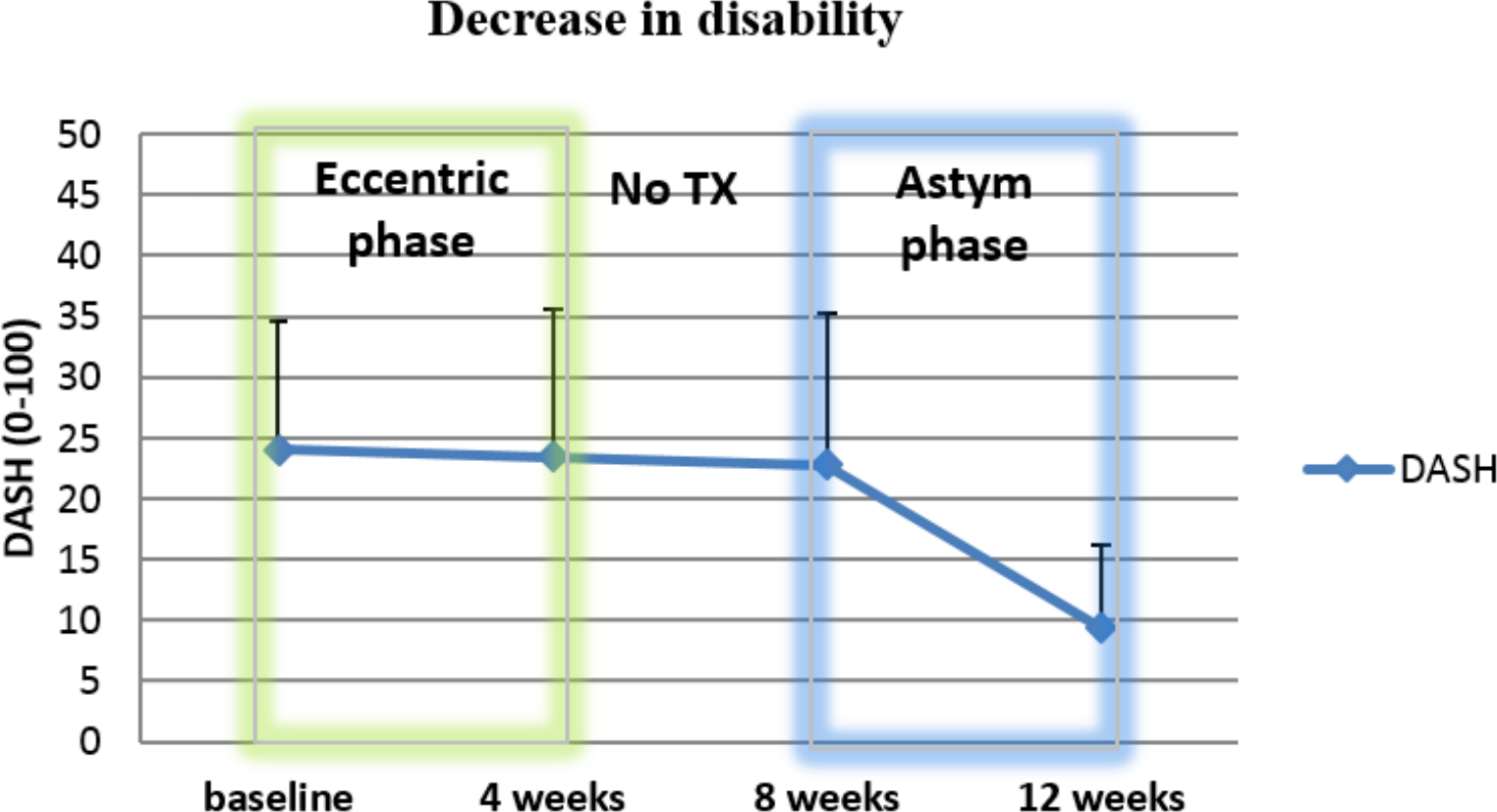
Mean and standard deviations of DASH scores for the Astym Delayed Entry Group. Astym treatment started at 8 weeks for recalcitrant eccentric subjects who opted to receive Astym therapy.

After the Astym intervention (8–12 week time period) the Astym Delayed Entry Group (*n* = 21) responded by showing 58.8% improvement in DASH scores, a 26.1% improvement with pain during activity, an 11.0% improvement of maximum grip strength, and a 24.3% improvement in the perceived function score. Table 4 shows the results of the one way ANOVA analyses for the baseline, 4 week, 8 week, and 12 week measurements. Figure 4 shows the means and standard deviations of the primary outcome measure, the DASH, over time.

The primary outcome measure, the DASH scores improved significantly over time (*p* < 0.005). From the secondary measures, pain with activity (*p* < 0.005) and function (*p* = 0.002) improved over the entire time frame for the Astym Delayed Entry subjects. Contrast analyses for each time frame (0–4 weeks, 4–8 weeks, and 8–12 weeks) summarized in Table 4, showed that the Astym Delayed Entry subjects made their improvements in the period from 8–12 weeks, the period in which they received Astym treatment.

Following Astym treatment, the Astym Delayed Entry Group subjects made gains similar to the subjects who received Astym treatment in the randomized experimental phase of the study. The experimental group DASH gain after 4 weeks of Astym treatment (mean 13.3; sd 12.9) was not significantly different (*p* = 0.96) from the DASH gain at 12 weeks for the Astym Delayed Entry Group (mean 13.4; sd 9.5). The randomized experimental group VAS active gain after 4 weeks of Astym treatment (mean 24; sd 27) was not significantly different (*p* = 0.48) from the VAS active gain at 12 weeks for the Astym Delayed Entry Group (mean 29; sd 30). The randomized experimental group function gain after 4 weeks of Astym treatment (mean 15; sd 28) was not significantly different (*p* = 0.54) from the function gain at 12 weeks for the Astym Delayed Entry Group (mean 19; sd 19). The experimental group maximum grip strength gain after 4 weeks of Astym treatment (mean 9.4 lbs; sd 18.5) was not significantly different (*p* = 0.66) from the maximum grip strength gain at 12 weeks for the Astym Delayed Entry Group (mean 5.8 lbs; sd 29.8 lbs).

***3. Long term outcomes***

Sixty eight of the 107 subjects (64%) returned follow up, mailed questionnaires. Subjects who returned questionnaires showed significant improvements on DASH scores, pain with activity, and function at six and 12 months as compared to baseline measurement (*p* < 0.001). There was no difference in long term gains between subjects who received Astym treatment initially in the randomized experimental period and those who received Astym treatment after week 8 in the Delayed Entry Group (DASH at 6 months, *p* = 0.58; DASH at 12 months, *p* = 0.76; VAS active at 6 month, *p* = 0.06; VAS active at 12 months, *p* = 0.21). No adverse effects were reported in either group.

## Discussion

Both comparative treatments in this study aim to improve the pathologic condition of the tendon, have evidence indicating positive physiologic effect (Davidson et al., 1997; Gehlsen, Ganion & Helfst, 1999; Murtaugh & Ihm, 2013), and have shown effectiveness in treating tendinopathy (Croisier et al., 2007; McCormack, 2012; Murtaugh & Ihm, 2013; Slaven, 2014; Wilson et al., 2000). Eccentric exercise has been suggested to be the first-line treatment for lateral elbow (LE) tendinopathy (Kaux et al., 2011). This study compares Astym therapy to an evidence-based eccentric exercise program (EE) in the treatment of LE tendinopathy.

The results show that subjects who received Astym therapy reported greater reductions in disability (DASH) and greater gains in maximum grip strength than subjects in the EE group. In 21 recalcitrant subjects who failed to respond to the initial EE program, four weeks of delayed entry Astym therapy resulted in statistically significant improvements in DASH scores, pain with activity and function. Long-term follow-up revealed that the subjects maintained their gains at 6 and 12 months, and these delayed entry subjects made gains similar to the gains made by patients who received Astym therapy as an initial treatment.

Responder information indicated that 78.3% of subjects who received Astym therapy resolved their symptoms, and 40.9% of subjects in the EE group resolved their symptoms in the initial 4 weeks of random assigned treatment. Of the subjects who showed recalcitrant tendinopathy by not resolving after the completion of the EE program, 95.7% achieved resolution following 4 weeks of Astym treatment. In total, 83.6% of all subjects who were treated with Astym therapy either initially or upon delayed entry met resolution criteria.

Although no consensus exists about the cut-off point for clinically relevant effect size (Ruyssen-Witranda, Tubacha & Ravauda, 2011), the observed effect size fall within the range of accepted clinically relevant effect sizes. The effect sizes for the initial treatment was 0.43 for the DASH, the primary outcome measure and ranged from 0.14 (function) to 0.62 for maximum grip strength. For the delayed entry group of the recalcitrant patients who received Astym treatment after failing to respond to the EE treatment effect sizes for change over time in ranged between 0.4 and 0.7, which considered a medium to large effect size (Cohen, 1988; Raman, MacDermid & Grewal, 2012). The treatment effect of Astym therapy on recalcitrant tendinopathy of the lateral elbow is encouraging for the many patients who struggle long term with the symptoms and functional limitations associated with chronic tendinopathy of the lateral elbow. In all Astym subjects (early and delayed), improvements over baseline continued at 6 and 12 months. None of the subjects reported any adverse effects.

The findings from the current study wherein a high percentage of the recalcitrant cases of lateral elbow tendinopathy resolved their symptoms after Astym treatment, may add support to other treatment methods which are designed proactively to influence intra-tendinous factors of tendon healing. Basic science indicates that the Astym process relies in part upon targeting cellular mediators and growth factors to assist in the regeneration of soft tissues (Davidson et al., 1997; Davies & Brockopp, 2010; Gehlsen, Ganion & Helfst, 1999; McCormack, 2012; Slaven, 2014). Similarly, Creaney et al. (2011) hypothesized that attempting to stimulate tissue regeneration through the delivery of growth factors could improve healing in patients with recalcitrant elbow tendinopathy who were resistant to conservative physical therapy consisting of stretching and eccentric exercise. Such a population of recalcitrant elbow tendinopathy was presumed to be difficult to treat. Creaney et al. (2011) administered platelet-rich plasma (PRP) injections (*n* = 80) and autologous blood injections (ABI) (*n* = 70) to separate portions of the subjects with recalcitrant elbow tendinopathy, which resulted in a 66% success rate for the PRP group and a 72% success rate for the ABI group.

Consistent with Creaney’s hypothesis that efforts to stimulate tissue regeneration may improve outcomes in recalcitrant elbow tendinopathy, 95.7% (20/21) of the recalcitrant cases of lateral elbow tendinopathy resolved after Astym treatment in this current study. In this study, the resolution rates of Astym treatment for recalcitrant tendinopathy exceed those of PRP and ABI in the Creaney study. Future studies need to be conducted to determine whether this difference may be explained by the more comprehensive nature of the Astym treatment approach, that intends to go beyond increasing endogenous growth factors and cellular mediators by: (1) including methods to locate and reduce fibrosis that may be contributing to the pathology; (2) the incorporation of stretching and strengthening components, and (3) being part of a larger rehabilitation approach designed to address the entire kinetic chain (Davies & Brockopp, 2010; McCormack, 2012; Slaven, 2014). Further study is also needed to determine whether the addition of these elements would improve the outcomes of PRP and ABI.

The results of this study may indicate that Astym treatment is in accordance with suggested criteria for the treatment of tendinopathy. In their review of the future of treatment of tendinopathy, Sharma and Maffulli stated, “the ideal treatment should accomplish its goal in a relatively short period of time with little discomfort or disability to the patient” (Sharma & Maffulli, 2008). Consistent with this proposal, the course of Astym treatment is typically four weeks or less, during which time the patient is encouraged to be active and continue with normal activities, preventing the consequences of imposed activity restriction. The goals of Astym treatment are also consistent with the objectives that they set out for tendinopathy treatments which they say should aim “to stimulate a healing response to restore the normal properties of tendons” (Sharma & Maffulli, 2008). These factors, combined with the results of this study, make Astym treatment appear to be a promising treatment for lateral elbow tendinopathy.

### Limitations

The study had a single blinded randomized design. All assessments were performed by physicians who were blinded to the patient group assignment, and the clinician taking measurements was also blinded. However, due to the nature of the treatments, it was not possible to blind the treating clinicians or the subjects.

The possibility of placebo effects was present in the design: the Astym treatment group was seen in the clinic twice per week and experienced a hands-on treatment. To counteract some of the placebo effect, subjects in the EE program had unlimited access to the treating EE clinician via phone, and EE clinician also initiated contact with the subjects on a regular basis. Even though EE subjects met with a clinician initially to receive exercise instruction and guidance on the exercise program, the subjects were not supervised during the actual performance of the exercise, which could have resulted in improper performance or non-compliance, however, no such concerns were reported. Although the optimal parameters of an eccentric exercise program have yet to be defined, additional exercise may carry additional benefit. If that is the case, the exercise component of Astym treatment may have been responsible for or contributed to the increased symptom relief experienced by the crossover patients who received Astym treatment after eccentric exercise. The Astym treatment group contained a higher number of male subjects with the EE group showing a larger proportion of females. Statistical analyses indicated that gender did not interact with the study results. Like most other lateral elbow tendinopathy studies, this study lacked a true control group of subjects not receiving any form of treatment. Therefore, one can argue that the observed difference between the two randomized treatment groups may indicate efficacy of the Astym over and above an exercise program. This design included a clinically relevant treatment strategy to accommodate the lives of subjects with long standing elbow tendinopathy who were motivated to receive care aimed at resolving their symptoms. The subjects followed a real life clinical treatment, in that they were discharged when symptoms abated. Therefore, the experimental treatment lasted 4 weeks, and after 8 weeks, the non-responders in the EE group were allowed to receive Astym treatment. Although the effects of NSAIDs on tendon healing are unclear, the medication has been suggested to have a detrimental effect under certain circumstances. Both groups were permitted to take 200 mg of the NSAID ibuprofen as needed for pain during the first two weeks of this study, which may have impacted their recovery.

Future research is needed to confirm the findings in larger randomized trials.

## Conclusion

Lateral elbow tendinopathy, or tennis elbow, although commonly diagnosed, is often considered difficult to treat. Prolonged symptoms and relapse are frequently observed. Eccentric exercise programs have been suggested to be a first-line of conservative treatment for tennis elbow, and have demonstrated encouraging results.

Astym treatment is a relatively new, non-invasive, conservative therapeutic approach that addresses tendinopathy by relying in part on the use of cellular mediators and growth factors to assist in the healing and regeneration of tissues.

This study compared Astym treatment to an evidence-based eccentric exercise program as suggested by Nirschl and Kraushaar (EE) in the treatment of tennis elbow. The results show that subjects who received Astym treatment reported greater reductions in disability (DASH) and greater gains in maximum grip strength than EE subjects. Subjects who failed to respond to the EE program, and were allowed delayed access to Astym therapy, showed statistically significant improvements in DASH scores, pain with activity and function. Long-term follow-up revealed that the subjects maintained their gains at 6 and 12 months, and these delayed entry subjects made gains similar to the gains made by patients who received Astym as an initial treatment. In the initial randomized group, 78% of subject resolved their symptoms with Astym treatment. The delayed entry group achieved a 95.7% resolution rate. In total, 83.6% of all subjects who were treated with Astym therapy either initially or upon delayed entry met resolution criteria.

The results of this study appear to indicate that Astym therapy is an effective conservative treatment option both in the initial management of lateral elbow tendinopathy, and in the management of recalcitrant, chronic lateral elbow tendinopathy. Additional randomized studies in multiple treatment settings are needed to substantiate these findings, and develop patient characteristic criteria of who may benefit most from this non-invasive treatment.

## Supplemental Information

10.7717/peerj.967/supp-1 Supplemental Information 1 CONSORT Checklist

10.7717/peerj.967/supp-2 Supplemental Information 2 Subject Raw Data

## Additional Information and Declarations

### Clinical Trial Registration

The following information was supplied regarding Clinical Trial registration: The study was conducted prior to registration requirement.
